# Therapeutic potential of Trichostatin A to control inflammatory and fibrogenic disorders of the ocular surface

**Published:** 2010-12-31

**Authors:** Ai Kitano, Yuka Okada, Osamu Yamanka, Kumi Shirai, Rajiv R. Mohan, Shizuya Saika

**Affiliations:** 1 Department of Ophthalmology, Wakayama Medical University, Wakayama, Japan; 2 Mason Eye Institute, University of Missouri-Columbia, Columbia, MO

## Abstract

**Purpose:**

To examine the effects of a histone deacetylase inhibitor, Trichostatin A (TSA), on the behavior of macrophages and subconjunctival fibroblasts in vitro and on ocular surface inflammation and scarring in vivo using an alkali burn wound healing model.

**Methods:**

Effects of TSA on expression of inflammation-related growth factors or collagen I were examined by real-time RT–PCR or immunoassay in mouse macrophages or human subconjunctival fibroblasts. Effects of TSA on trans forming growth factor β (TGFβ)/Smad signaling were evaluated with western blotting and/or immunocytochemistry. Alkali-burn injuries on the eyes of mice were performed with three µl of 0.5 N NaOH under general and topical anesthesia. TSA (600 µg/Kg daily) or vehicle was administered to animals via intraperitoneal (i.p.) injection. Histology and real-time RT–PCR investigations evaluated the effects of TSA on the healing process of the cornea.

**Results:**

TSA inhibited TGFβ 1 and vascular endothelial growth factor (VEGF) expression in macrophages, and TGFβ1 and collagen I in ocular fibroblasts. It elevated the expression of 5′-TG-3′-interacting factor (TGIF) and Smad7 in fibroblasts and blocked nuclear translocation of phospho-Smad2. Real-time PCR and immunocytochemistry studies showed that systemic administration of TSA suppressed the inflammation and fibrotic response in the stroma and accelerated epithelial healing in the alkali-burned mouse cornea.

**Conclusions:**

Systemic administration of TSA reduces inflammatory and fibrotic responses in the alkali-burned mouse ocular surface in vivo. The mechanisms of action involve attenuation of Smad signal in mesenchymal cells and reduction in the activation and recruitment of macrophages. TSA has the potential to treat corneal scarring in vivo.

## Introduction

Fibroblasts and macrophages induce inflammatory and/or fibrogenic disorders in various tissues by expressing profibrogenic cytokines and/or extracellular matrix (ECM) components [1]. Pro-inflammatory cytokines expressed by these cell types are a further chemoattractant to inflammatory cells. Among ocular surface fibrogenic diseases, alkali burn, vernal or atopic conjunctivitis, and Stevens-Johnson’s syndrome are common [1,2]. Inflammatory reaction in the ocular surface causes activation of subconjunctival fibroblasts followed by fibrogenic sequealae and potentially leads to visual impairment by damaging the ocular surface.

The profibrogenic phenotype of tissue mesenchymal cell types could be modulated by a combination of epigenetic alterations such as methylation and (de)acetylation which are reversible, and offer a potential opportunity to reverse the epigenetic pattern [3,4]. In normal resting cells, DNA is organized within nucleosomes in chromatin and proteins, histones, that regulate the level of gene transcription [5]. Histone hyperacetylation generally promotes gene transcription. Deacetylation of histones mediated by histone deacetylases (HDACs) causes wrapping of the DNA around the nucleosome and prevents transcription factors from binding to it [6]. HDACs are enzyme complexes that remove the acetyl group from the histones [7]. Trichostatin A (TSA, m.w.=302.4) is a potent reversible HDAC inhibitor [8]. TSA has been tested in clinical trials for cancer therapy based on its effect of cell cycle arrest [9-11] and also has been considered a potential therapeutic agent against fibrogenic diseases likes hepatic fibrosis and cutaneous radiation syndrome [12-15]. Although the mechanism is not fully understood, it might include suppression of Smad-mediated gene expression by TSA. In ocular surface tissues (cornea or conjunctiva), we recently reported that TSA suppresses myofibroblast generation, one of the hallmarks of fibrosis, in cultured keratocytes and significantly reduces stromal haze in the rabbit cornea following excimer laser injury [16]. However, effects of systemic administration of a TSA on inflammation-related fibrogenic reaction in the ocular surface, and its effects on pro-inflammatory cytokine expression in the macropahges, and on signal transduction in ocular fibroblasts have not yet been investigated.

In the present study, we tested the effects of TSA on (i) fibrogenic behavior i.e., proliferation, migration, expression of fibrogenic mediators, etc.; (ii) signal transduction of cultured human subconjunctival fibroblasts; and (iii) inflammatory reaction in cultured macrophages. Furthermore, we investigated whether systemic administration of TSA has a therapeutic effect on ocular surface fibrosis using an alkali-burn mouse model. The goal of the current study was to evaluate the therapeutic potential of TSA in patients with ocular surface inflammatory fibrogenic diseases of the ocular surface.

## Methods

### Primary subconjunctival fibroblast culture

Human subconjunctival fibroblasts were cultured as described previously [17]. In brief, subconjunctival tissue was obtained during strabismus surgery with informed consent from the patients’ parents. The cells were cultured for 2 or 3 passages in Eagle’s minimum essential medium (MEM; Gibco, Grand Island, NY) supplemented with antibiotics, an antimycotic, and 10% fetal calf serum (MEM-10) before the following experiments. A stock solution of TSA (Sigma, St. Louis, MO) at a concentration of 2 M [18] was prepared in ethanol and stored at −80 °C. The final concentration of ethanol in the medium was 0.06%.

### Cell migration

Cell migration was examined by scratch assay as reported previously [19]. In brief, closure of a liner defect produced in the cell monolayer was determined in the presence or absence of TSA (10, 200, and 500 nM) at 36 h post scratching.

### Quantification of mRNA by real-time PCR

Confluent ocular surface fibroblasts in a 60-mm tissue culture dish were generated and incubated for 24 h in the presence or absence of various concentrations of TSA in serum-free medium. Total RNA was extracted using a Mammalian Total RNA Miniprep Kit (Sigma, St. Louis, MO) as reported earlier [19,20]. The expression of transforming growth factor β1 (*TGFβ1*) and collagen Iα1 mRNA was determined with real-time RT-PCR using appropriate TaqMan probes (Applied Biosystems). As for this probe, forward primer and reverse primer were already done premixing of. The real-time RT-PCR method using the Taqman One-Step RT-PCR Master Mix Reagents Kit and the Applied Biosystems Prism 7300 (P-E Applied Biosystems, Foster City, CA) was employed. The RT-PCR conditions were as follows: 30 min at 48 °C (stage 1, reverse transcription), 10 min at 95 °C (stage 2, reverse transcription inactivation and AmpliTaq Gold activation), and then 40 cycles of amplification for 15 s at 95 °C and 1 min at 60 °C (stage 3, polymerase chain reaction).

### ELISA assay

ELISA assays were performed to determine the expression TGFβ1 and fibronectin protein levels in subconjunctival fibroblasts using an ELISA kit purchased from R&D Systems (Minneapolis, MN). Cultures grown in a 24-well culture dish were incubated in the presence or absence of TSA in 1 ml serum-free medium for 48 h. Culture medium was harvested, and stored at −80 °C. The protein levels in samples were measured with an ELISA kit following the manufacturer’s instructions as described previously  [21,22]. We evaluated protein levels after having reacted reagent and samples by measuring absorbance.

### Immunocytochemistry

The cells (1.8×10^3^ in 300 µl medium/well) were grown to subconfluency in each well of 8-well Nunc Lab-Tec chamber slides (Nunc, Rochester, NY) and then grown in the presence or absence of TSA in serum-free medium for 24 h. The cultures were washed and fixed with cold acetone. Immunohistochemistry for collagen type I and fibronectin (EDA domain) protein was performed using goat polyclonal anti-collagen I (1:100 dilution in PBS; Southern Biotechnology, Birmingham, AL) and goat polyclonal anti-fibronectin (1:100 dilution in PBS; Santa Cruz Biotechnology, Santa Cruz, CA) antibodies, respectively, followed by the incubation with FITC-conjugated secondary antibodies (1:100 dilution in PBS; ICN Biomedicals, Aurora, OH) and nuclear staining with DAPI dye following the method reported earlier [19,20,23]. The specimens were observed under fluorescent microscopy.

### Effects of TSA on cell signal transduction

The effects of TSA on TGFβ1/Smad signaling were evaluated using western blot and immunocytochemistry techniques. For western blot analysis, subconjunctival fibroblasts were grown to confluence in 60-mm culture dishes, and then treated with various concentrations (up to 500 nM) of TSA for 24 h. Cultures were then exposed to exogenous TGFβ1 for 1 or 2 h, harvested in Sigma Mammalian Cell Lysis buffer (100 µl/dish, Sigma-Aldrich, St. Louis, MO), and subjected to SDS–PAGE and western blotting [24]. Polyclonal primary antibodies against phospho-Smad2 (pSmad2; Chemicon International, Temecula, CA), Smad2 (Santa Cruz Biotechnology), TGIF (Santa Cruz), Smad7 (Santa Cruz), and β-actin (Santa Cruz) were used. Smad2 is a TGFβ signal transducer and is activated by phosphorylation. Smad7 is an inhibitory Smad that blocks phosphorylation and nuclear translocation of Smads2/3. TGIF is also a negative regulator of TGFβ/Smad signal.

For immunocytochemistry, subconjunctival fibroblasts (4.0×10^3^/well in 300 µl medium) were grown to subconfluence in 8-well Nunc Lab-Tec chamber slides., Cultures were grown in the presence or absence of TSA in serum-free medium for 6 h after growing them in MEM-10 for 24 h. Then, cultures were treated with TGF β 1 (1.0 ng/ml, R&D Systems) for 0.5, 1, or 2 h. Samples were washed, fixed with 4% paraformaldehyde, and processed for immunocytochemistry for phosphorylated Smad2 (pSmad2) using anti-phospho-Smad2 antibody (Chemicon) [25].

### Primary macrophage culture

Mouse macrophages were obtained from the peritoneal space using a glycogen stimulation method as previously reported [19]. In brief, 5% sterilized oysterglycogen (Sigma-Aldrich) was injected into the peritoneal space of mice. After 4 days, the peritoneal cavity was irrigated with culture medium to harvest macrophages. About 90% of cells were positive for F4/80. F4/80 is a transmembrane protein present on the cell-surface of mouse macrophages. Markers of mouse macrophage development detected by moncolonal antibodies. The expression of *TGFβ1* and *VEGF* mRNA was determined with real-time RT-PCR using appropriate TaqMan probes (Applied Biosystems) as reported earlier [20].

### In vitro neovascularization

A commercial kit containing an in vitro co-culture system of human vascular endothelial (HUVECs) and fibroblast cells was used according to the manufacturer’s instructions (NV kit; Kurabo, Tokyo, Japan). This system co-cultures vascular endothelial cells on a fibroblast feeder layer and is used to evaluate new vessel formation based on increases in CD31-positive tube-like tissue formation. The effect was determined by adding TSA (10, 20, and 500 nM) on VEGF-A (10 ng/ml; Kurabo, Tokyo, Japan) stimulated vessel-like tube formation according to the protocol provided by the manufacturer. Tube-like tissue was detected by immunostaining for CD31, an endothelial cell marker at day 11 of culture. Color development was performed by diaminobenzidine (DAB) color reaction as reported [19]. Five wells were prepared for each culture condition. The length and number of branch points and the mean value was determined in three different 300 mm^2^ regions.

### Effect of TSA on ocular surface inflammation and fibrosis in an alkali-burn mouse model

We finally examined the therapeutic effects of systemic administration of TSA on the ocular surface fibrosis induced in mice by the topical application of 0.5N NaOH. The study was approved by the Institutional Animal Care and Use Committee, and animals were treated in accordance with the tenets of the ARVO Statement for the Use of Animals in Ophthalmic and Vision Research. NaOH solution (3 µl of a 0.5N stock) was topically applied onto the eye of adult C57BL/6 mice (n=44) under general (ketamine hydrochloride 40 mg/kg and xylazine hydrochloride 5 mg/kg from Sigma) and topical (1 drop of proparacaine hydrochloride 0.5% from Bausch and Lomb, Pharmaceuticals, Inc. Tampa, FL) anesthesia 24 h after i.p. injection of TSA (600 µg/kg, n=22) or vehicle (n=22). The mice continued to receive TSA (600 µg/kg) or vehicle through i.p. injection until euthanasia. The TSA-treated and control animals were sacrificed either on day-5 (n=12) or day-10 (n=10) after imaging the eyes. The eyes were fixed in 4% paraformaldehyde and processed for paraffin sections.

Immunohistochemistry was conducted using antibodies against ”α smooth muscle actin (αSMA), F4/80, or collagen IV (α1chain; a polyclonal antibody from Southern Biotechnology, Temecula, CA) as previously reported [25,26].

Another set of similar experiments with TSA (n=20) and control (n=20) was performed to evaluate *αSMA* (myofibroblast appearance), *F4/80* (macrophage invasion), *TGFβ1*, and collagen type I expression using real-time RT–PCR. The animals were sacrificed at day-5 or day-10 and their corneas were excised. The total RNA extraction was performed using two corneal tissues for one reaction. Real-time RT–PCRs were performed following the methods reported earlier [16,20,27]. The real-time RT-PCR method using the Taqman One-Step RT-PCR Master Mix Reagents Kit and the Applied Biosystems Prism 7300 (P-E Applied Biosystems, Foster City, CA) was employed.

## Results

### Effects of TSA on fibrogenic gene expression of subconjunctival fibroblasts

**Figure 1:**
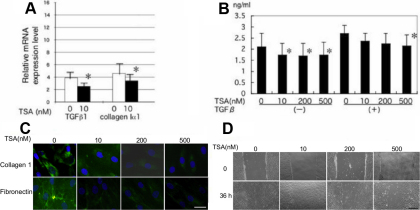
Effect of Trichostatin A (TSA) on the expression of fibrogenic components in cultured human subconjunctival fibroblasts. **A**: Real-time RT–PCR showed that 10 nM of TSA suppressed mRNA expression of transforming growth factor β1 (*TGFβ1*) and collagen I a1 chain. **B**: TSA reduced the production of TGFβ1 at the protein level in the presence of exogenous TGFβ1. *p<0.05. **C**: Immunocytochemistry showed reduction of protein expression of collagen type I in the cell cytoplasm and deposition of fibronectin in the cell layer by adding concentrations of TSA. Bar, 100 µm. **D**: Effects of TSA on fibroblast migration were evaluated by scratch assay. A liner a defect was produced in a fibroblast monolayer. The cells migrated into the defect and the defect was closed in 36 h in the control culture. TSA retarded migration of fibroblasts into the defect. Bar, 200 µm.

Real-time RT–PCR showed TSA suppressed mRNA expression of *TGFβ1* and collagen Iα1 in fibroblast culture (Figure 1A). ELISA also showed that TSA reduces the secretion of TGFβ1 protein (Figure 1B). Immunohistochemistry showed that TSA decreased the degree of staining for type I collagen in the cell cytoplasm as well as deposition of fibronectin EDA domain in the cell layer (Figure 1C).

### Fibroblast proliferation and migration

Addition of TSA to culture suppressed cell migration in a dose-dependent manner is determined by scratch assay (Figure 1D). Migration of cells into the defect was impaired relatively more at higher doses compared to that at lower doses of TSA.

### Effects of TSA on TGFβ1 signal transduction

**Figure 2:**
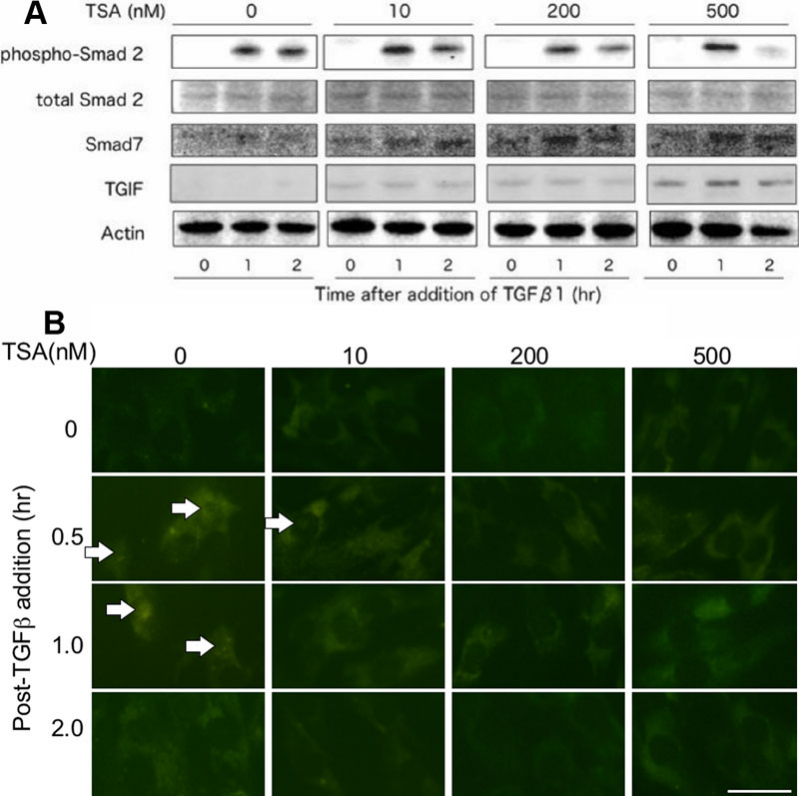
Effect of Trichostatin A (TSA) on expression of transforming growth factor β1 (TGFβ1)-related signal transduction in cultured human subconjunctival fibroblasts. **A**: western blotting indicates that at 2 h post-TGFβ1 addition TSA at concentrations of 200 nM faintly reduced the level of phosphorylated Smad2 (pSamd2) as compared with the phosphorylation level at 1 h post-TGFβ1 addition, while the cells maintained a similar level of phosphorylation of Smad2 until 2 h in the absence of TSA. TSA at a concentration of 500 nM obviously reduced its phosphorylation level at 2 h. Adding TSA upregulated the expression of Smad7 and TGIF in a dose-dependent manner. **B**: Immunocytochemistry also showed that TSA suppressed nuclear translocation of pSmad2. In the absence of TSA pSmad2 was detected in cell nuclei at 0.5 and 1.0 h post-TGFβ1 addition. With TSA at a concentration of 10 nM pSmad2 was detected in the nuclei after 0.5 h. TSA at the concentrations of 200 and 500 nM abolished its nuclear expression.

As for the TGFβ1/Smad signal, the level of phospho-Smad2 2 h post-TGFβ1 addition TSA at a concentration of 200 nM faintly reduced compared with the phosphorylation level 1 h post-TGFβ1 addition. While the cells maintained a similar level of phosphorylation of Smad2 until 2 h in the absence of TSA. TSA at a concentration of 500 nM substantially reduced the Smad2 phosphorylation level at 2 h. The addition of TSA to cultures upregulated the expression of Smad7 and TGIF in a dose-dependent manner (Figure 2A).

Immunocytochemistry also showed that TSA suppressed nuclear translocation of pSmad2 (Figure 2B). In the absence of TSA, pSmad2 was detected in cell nuclei at 0.5 and 1.0 h post-TGFβ1 addition (Figure 2B); whereas in the presence of TSA (10 nM), phospho-Smad2 was detected in the nuclei after 0.5 h. TSA at the concentrations of 200 and 500 nM abolished pSmad2 nuclear expression (Figure 2B).

### Cytokine expression by macrophages

**Figure 3:**
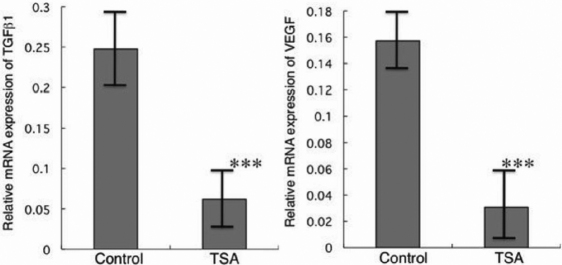
Effects of TSA on cytokine expression in cultured macrophages. Expression of transforming growth factor β1 (*TGFβ1*) and vascular endothelial growth factor (*VEGF*) mRNAs in cultured macrophages incubated for 24 h in the presence or absence of 10 nM TSA as evaluated by real-time RT–PCR. Expressions of *TGFβ1* and *VEGF* were markedly suppressed by continuous exposure to TSA. ***p<0.01.

The RT–PCR studies demonstrated that macrophages exposed to 10 nM of TSA for 24 h significantly suppressed levels of *TGFβ1* and *VEGF* mRNA expression (Figure 3). The continuous exposure of TSA to other tested higher concentrations showed more pronounced effects (data not shown).

### In vitro neovascularization

**Figure 4:**
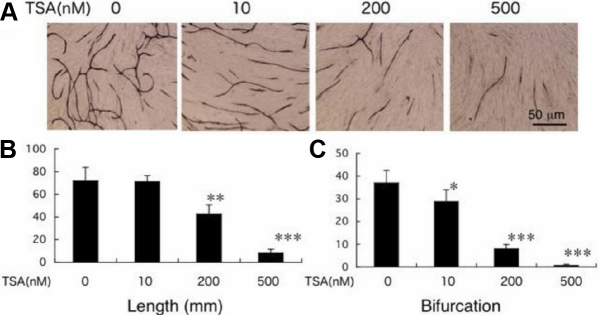
Effects of Trichostatin A (TSA) on in vitro neovascularization were evaluated by a co-culture model of human umbilical vein endothelial cells (HUVECs) and fibroblasts. **A**: HUVECs form CD31-positive tube-like structure on a fibroblast feeder layer in the presence of vascular endothelial growth factor. TSA seems to inhibit formation of CD31-labeled tube-like tissues. Bar, 50 mm. **B**: When evaluated by the measurement of total length and the number of the branching points of vessel-like tubes formed by HUVECs, TSA at the concentrations inhibited elongation of CD31-labeled tube like formation by HUVECs in a dose-dependent manner. *, **, and *** represent p<0.05, 0.01, and 0.005, respectively.

To determine the effects of TSA on corneal neovascularization, HUVEC cells were grown on the fibroblast feeder layer. HUVECs form CD31-positive tube-like structures on a fibroblast feeder layer in the presence of VEGF. When evaluated by the measurement of total length and the number of the branching points of vessel-like tubes formed by HUVECs, TSA at the tested concentrations inhibited elongation of CD31-labeled tube-like structures in a dose-dependent manner (Figure 4). All of the three tested doses (10, 200, and 500 nM) of TSA significantly inhibited bifurcation and branching of the tubes. Except for the10 nM dose of TSA, all other tested doses similarly showed significant reduction in the length and density of these tube-like structures.

### Evaluation of the effect of systemic TSA on ocular surface inflammation and fibrosis caused by an alkali burn in mice

**Figure 5:**
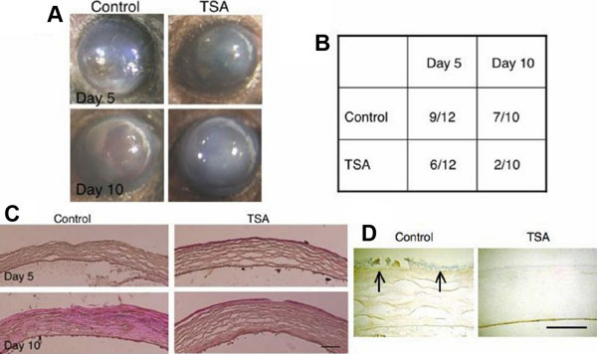
Evaluation of the effect of systemic Trichostatin A (TSA) on ocular surface fibrosis model of an alkali burn in mice. **A**: An alkali burn of the ocular surface resulted in epithelial defects, stromal scarring (opacification) and neovascularization on day 5 and day 10. Treatment of the animals with systemic TSA, i.p., seemed to reduce the incidence of residual epithelial defects and stromal opacification. **B**: The incidence of residual epithelial defects was significantly less ons day 10, but not on day 5, as evaluated using the χ^2^-square test (p<0.05). **C**: Histology by hematoxylin and eosin staining shows that alkali exposure induced inflammation with epithelial defects in the stroma. More cellularity, presumably due to inflammatory cells, was seen in control animals compared with that in TSA-treated mice on days 5 and 10. **D**: Irregular accumulation of type VI collagen α1chain was detected (arrows) in the stroma beneath the healing epithelium in a control animal, while it was not detected in the well healed epithelium of a cornea from the TSA group. Bar, 100 µm.

An alkali burn of the ocular surface resulted in corneal epithelial defect in the early phase (day 5) and then stromal scarring (opacification) and neovascularization in the later phase (day 10; Figure 5A). Systemic TSA treatment (600 µg/kg/day; i.p.) given to mice markedly reduced the incidence of epithelial defect at day-10 in the post-alkali burn eyes as demonstrated by the χ^2^-square test (Figure 5B).

Histological results coincided with the findings from ocular surface observation. H&E staining showed that alkali exposure induced inflammation with epithelial defects in stroma. Increased cellularity, presumably inflammatory cells, was seen in control corneal sections (Figure 5C; left panels) compared to that in TSA-treated corneal sections (Figure 5C; right panels). Immunohistochemistry also showed an irregular accumulation of type IV collagen α1 peptide in the stroma beneath the healing epithelium in a control animal, while it was not detected in the well healed epithelium of a TSA group cornea (Figure 5D).

**Figure 6:**
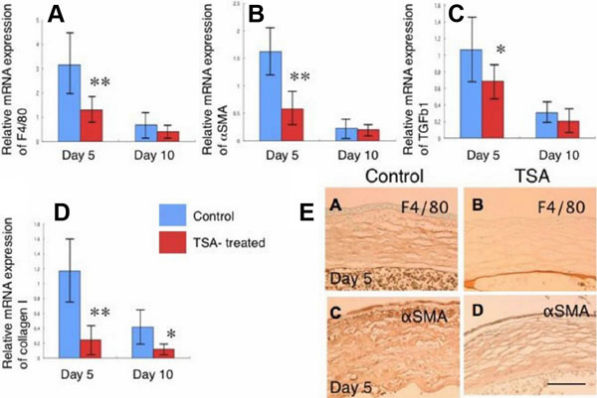
Characterization of healing of an alkali-burned cornea treated with systemic Trichostatin A (TSA) by real-time reverse transcription polymerase chain reaction (real-time RT–PCR) and immunohistochemistry. Real-time RT–PCR showed that systemic TSA treatment suppressed mRNA expression levels of a macrophage antigen, F4/80 (**A**), α-smooth muscle actin (αSMA; **B**), transforming growth factor β1 (TGFβ1; **C**) and collagen typeI (**D**) in a healing alkali-burned mouse cornea on day 5 and day 10. **p<0.01, *p<0.05. **E**: Immunohistochemistry also showed fewew myofibroblasts and macrophages in a TSA-treated healing alkali-burned mouse cornea compared with those in control mice. Bar, 100 µm.

Real-time RT–PCR showed that systemic TSA treatment significantly suppressed mRNA expression levels of *F4/80*, *αSMA*, *TGFβ1*, and collagen type-I in the healing alkali-burned mouse corneas on day 5 or day 10 (Figure 6A-D). Immunohistochemistry analysis of corneal tissue sections showed fewer myofibroblasts and macrophages in TSA-treated (Figure 6E; right panels) healing alkali-burned mouse corneas than the control corneas (Figure 6E; left panels).

## Discussion

In the present study we showed that TSA exhibited anti-inflammatory as well as anti-profibrogenic activity in cultured macrophages or human subconjunctival fibroblasts. The TSA treatment suppressed the expression of TGFβ1 and VEGF in cultured macrophages and TGFβ1 and collagen type I as well as expression of αSMA, the marker for a myofibroblast, in cultured subconjunctival fibroblasts. We hypothesize that the suppression of TGFβ1 expression by TSA is due to the reduction of collagen in TSA-culture. Reduced deposition of fibronectin in cell layers might be caused by reduced collagen accumulation that reportedly forms a scaffold for fibronectin deposition in cell layer. Nonetheless, the addition of TSA did not affect cell proliferation (data not shown). Consistent with the data from other studies, our findings indicate involvement of epigenetic histone modifications in the expression of pro-inflammatory or profibrogenic components in the cornea. Similar in vitro findings of antifibrogenic effects of TSA were reported in cultured rat mesangial cells [28].

Several possible mechanisms for TSA-mediated inhibition of proinflammatory or profibrogenic response have been postulated. TSA or TSA-induced gene products are known to interfere with the signaling cascade(s) involved in cell proinflammatory and/or profibrogenic reaction. Binding of TGFβ to its receptor phosphorylates Smad2/3 and activates the signaling cascade. The phosphorylated Smad2 and Smad3 then form a complex with Smad4 and translocate to the nucleus [29,30]. Together with Smad co-activator(s) or co-repressor(s), the Smad2/3/4 complexes regulate the expression of several profibrogenic genes. In the present study, we examined expression patterns of Smad-related molecules by western blotting and immunocytochemistry. The results of our study show that TSA upregulates Smad7 (an inhibitory Smad that blocks phosphorylation of Smad2/3) and TGIF (a co-repressor of Smad-dependent gene expression that directly associates with Smad proteins) and inhibits Smad-mediated transcriptional activation in cultured subconjunctival fibroblasts.

Immunocytochemistry showed that even a low TSA dose (10 nM) suppressed translocation of pSmad2 to the nuclei whereas high doses of TSA (200 and 500 nM) eliminated the pSmad2 signal from the cell nuclei as no pSmad2 expression was detected in the cell nuclei at these 2 TSA doses. Western blot analysis of these experiments was also in agreement with the immunocytochemistry data, except the 200 nM dose of TSA. Western blot analysis of samples treated with 200 nM TSA did not completely block phosphorylation of Smad2; instead, it faintly reduced the pSmad2 levels at 2 h post-TGFβ1 addition compared to the Smad2 phosphorylation level noted 1 h post-TGFβ1 addition. The levels of phosphorylation of Smad2 in control samples were about the same at 1 and 2 h. The discrepancy noted for the 200 nM TSA concentration could not be completely explained at this time. We speculate that this might be due to Smad2 import in the nuclei as TSA not only reduces the phosphorylation level of Smad2 but also inhibits its import to the nuclei. Moreover, it is also likely that upregulation of TGIF might have suppressed the Smad-dependent gene expression at the gene promoter level.

Tissue scarring is well characterized by the invasion of inflammatory cells such as macrophages, and subsequent generation of myofibroblasts that pose αSMA cytoskeletal fibers from the fibroblasts. Expression of αSMA is regulated by the gene promoter activity and extracellular scaffold. The αSMA promoter is activated by the Smad2 but the appearance of αSMA contractile cytoskeletal fibers or generation of myofibroblasts depends on extracellular deposition of fibronectin, especially ED-A type fibronectin [31]. Thus, we postulate that suppression of myofibroblasts by TSA is due to the suppression of Smad activity by the upregulation of Smad7 and TGIF and inhibition of extracellular deposition of fibronectin.

Our in vitro studies led us to hypothesize that systemic administration of TSA might suppress the inflammatory fibrogenic reaction in an alkali-burned cornea. To test this hypothesis we conducted in vivo experiments using a mouse cornea-alkali burn model. The intraperitoneal administration of TSA was chosen over topical administration on the eye to avoid washing TSA from the ocular surface by the tears. However, therapeutic levels of TSA can be maintained in the eyes of patients via topical application of TSA. The results of histology, real-time RT–PCR, and immunohistochemistry experiments suggest that systemic TSA treatment exhibits therapeutic anti-inflammatory and antifibrogenic activities in an alkali-burned mouse cornea. Administration of systemic TSA treatment (i.p.) to the animals reduced the incidence of epithelial defect(s) and stromal inflammation. Evaluation of the gene expression pattern showed that systemic TSA inhibited the appearance of myofibroblasts, as detected by αSMA expression and macrophage invasion, as indicated by the expression of F4/80. Such phenomenon could explain the suppression of mRNA expression of *TGFβ1*, presumably expressed mainly in macrophages, and collagen I, a major fibrogenic product expressed by myofibroblasts, in a TSA-treated healing cornea. Besides histology, epithelial regeneration was further evaluated by immunodetection of type IV collagen α1 chain. Normal epithelial basement membrane of the cornea does not contain type IV collagen α1 chain dissimilar to the usual basement membrane, while this type of α chain appears in a newly regenerated immature basement membrane in an injured cornea [32-34]. Thus, the presence of α1chain of type IV collagen suggests unorganized regeneration of the healing basement membrane of the corneal epithelium. In the present study, the TSA-group cornea exhibited no immunolabeling of this type of α chain in the epithelial basement membrane, while the α1 chain was detected beneath the partially regenerated epithelium. This finding further supports the acceleration of restoration of normal corneal structure following TSA administration.

Our data are consistent with the results of previously published studies that demonstrated antifibrotic properties or anti-Smad3-driven epithelial-mesenchymal transition of TSA [13,35]. Similar in vivo findings showing that TSA suppresses macrophage invasion and local finbrogenic reaction were observed in an animal model of renal fibrosis [36]. Our preliminary experiment showed that there was such no beneficial effect of daily TSA, i.p., on the healing process in an alkali-burned mouse cornea at 1 month post-alkali treatment (data not shown). The exact reason why we did not observe the efficacy in the present alkali-burn model is also necessary further investigate whether this might be the case in other animals as well. Nevertheless, the present findings support the potential utility of the drug in treating human cases.

In conclusion, TSA has great potential to be an effective drug to treat inflammatory and fibrotic disorders in the ocular surface among patients. Mechanism of action might include suppression of cytokine expression, migration of macrophages, inhibition of TGFβ-driven fibroblast activation and/or myofibroblast conversion. Further study is needed to establish the clinical utility and the suitable route(s) of administration of the drug for the treatment of ocular surface inflammatory fibrogenic disorders.
